# VEGF/VEGFR-Targeted Therapy and Immunotherapy in Non-small Cell Lung Cancer: Targeting the Tumor Microenvironment

**DOI:** 10.7150/ijbs.70958

**Published:** 2022-05-29

**Authors:** Yueshui Zhao, Sipeng Guo, Jian Deng, Jing Shen, Fukuan Du, Xu Wu, Yu Chen, Mingxing Li, Meijuan Chen, Xiaobing Li, Wanping Li, Li Gu, Yuhong Sun, Qinglian Wen, Jing Li, Zhangang Xiao

**Affiliations:** 1 Laboratory of Molecular Pharmacology, Department of Pharmacology, School of Pharmacy, Southwest Medical University, Luzhou, Sichuan 646000, China; 2 Department of Oncology, The Affiliated Hospital of Southwest Medical University, Luzhou, Sichuan 646000, China; 3 Cell Therapy & Cell Drugs of Luzhou Key Laboratory, Luzhou, Sichuan 646000, China; 4 South Sichuan Institute of Translational Medicine, Luzhou, Sichuan 646000, China; 5 Department of Oncology and Hematology, The Affiliated Traditional Chinese Medicine Hospital of Southwest Medical University, Luzhou, Sichuan 646000, China; 6 Center of Excellence for Molecular Imaging (CEMI), Department of Radiologic Technology, Faculty of Associated Medical Sciences, Chiang Mai University, Chiang Mai, Thailand

**Keywords:** NSCLC, tumor microenvironment, VEGF/VEGFR pathway, immunotherapy, clinical trials

## Abstract

Non-small cell lung cancer (NSCLC) is the leading cause of death by cancer worldwide. Despite developments in therapeutic approaches for the past few decades, the 5-year survival rate of patients with NSCLC remains low. NSCLC tumor is a complex, heterogeneous microenvironment, comprising blood vessels, cancer cells, immune cells, and stroma cells. Vascular endothelial growth factors (VEGFs) are a major mediator to induce tumor microvasculature and are associated with the progression, recurrence, and metastasis of NSCLC. Current treatment medicines targeting VEGF/VEGF receptor (VEGFR) pathway, including neutralizing antibodies to VEGF or VEGFR and receptor tyrosine kinase inhibitors, have shown good treatment efficacy in patients with NSCLC. VEGF is not only an important angiogenic factor but also an immunomodulator of tumor microenvironment (TME). VEGFs can suppress antigen presentation, stimulate activity of regulatory T (Treg) cells, and tumor-associated macrophages, which in turn promote an immune suppressive microenvironment in NSCLC. The present review focuses on the angiogenic and non-angiogenic functions of VEGF in NSCLC, especially the interaction between VEGF and the cellular components of the TME. Additionally, we discuss recent preclinical and clinical studies to explore VEGF/VEGFR-targeted compounds and immunotherapy as novel approaches targeting the TME for the treatment of NSCLC.

## 1. Introduction

Lung cancer is a leading cause of cancer-related death, and non-small cell lung cancer (NSCLC) is the most common type of lung cancer 1. The prognosis of NSCLC remains poor, and the overall recovery and survival rates of patients remain low, as most patients with NSCLC have advanced cancer or extensive metastases before diagnosis 2-4. The tumor microenvironment (TME) of NSCLC is complex, comprising various types of immune cells and vasculature. Vascular endothelial growth factor (VEGF) is mainly secreted by the endothelial cells of blood vessels; however, it can also be generated by the immune cells of the TME, such as tumor-associated macrophages (TAM) 5, tumor-associated neutrophils (TAN) 6, 7, mast cells (MC) 8-10, myeloid-derived suppressor cells (MDSC) 11-13, and natural killer cells (NK) 14, 15. The VEGF is not only linked with multiple functions in angiogenesis but also suppresses immune cells and promotes local and systemic immunosuppression in cancer. In recent years, owing to the successful development of immune checkpoint inhibitors, vaccines, VEGF/VEGF receptor (VEGFR)-targeting compounds, and other immunotherapeutic agents for cancer treatment, a new approach to treat various tumors, which combines anti-angiogenic drugs with immunotherapy, has emerged 16. Emerging evidence demonstrates potential synergistic efficiency between VEGF/VEGFR-targeting compounds and immunotherapy for NSCLC treatment. In the present review, we focus on the angiogenic and non-angiogenic functions of VEGF in NSCLC, in particular, on the interaction between VEGF and the cellular components of the TME. In addition, we discuss recent preclinical and clinical studies to explore VEGF/VEGFR-targeted compounds and immunotherapy as novel approaches targeting the TME for NSCLC treatment.

## 2. Functions of VEGF/VEGFR in NSCLC

### 2.1 Angiogenic function: tumor vasculature

VEGF is mainly secreted by tumor cells, some stromal cells, and endothelial cells in the TME 17. The VEGF family comprises several members, including VEGF-A, VEGF-B, VEGF-C, VEGF-D, VEGF-E, VEGF-F, placental growth factor (PLGF), and endocrine gland-derived VEGF (EG-VEGF) 17, 18.

The members of the VEGF family perform their functions by binding with their receptors. VEGF receptors are categorized into two types: tyrosine kinase receptors (VEGF receptors, VEGFR), which include VEGFR-1, VEGFR-2, and VEGFR-3, and neuropilin receptors (NRPs), which include NRP-1 and NRP-2 19, 20. NRPs are the co-receptors of VEGF; the binding of VEGF and NRPs enhances the stability of the receptor complex 19.

The VEGF family members selectively bind to VEGFR. VEGF-A is the major member of the VEGF family for angiogenesis; it is expressed in all vascular tissues, macrophages, tumor cells, and other cells 21, 22. Moreover, it can bind to both VEGFR-1 and VEGFR-2, but it primarily binds to the latter for dimerization, autophosphorylation, and activation, thereby becoming crucial in downstream signaling, causing the proliferation and migration of endothelial cell, and performing angiogenic functions. 23-25.

**Figure 1:**
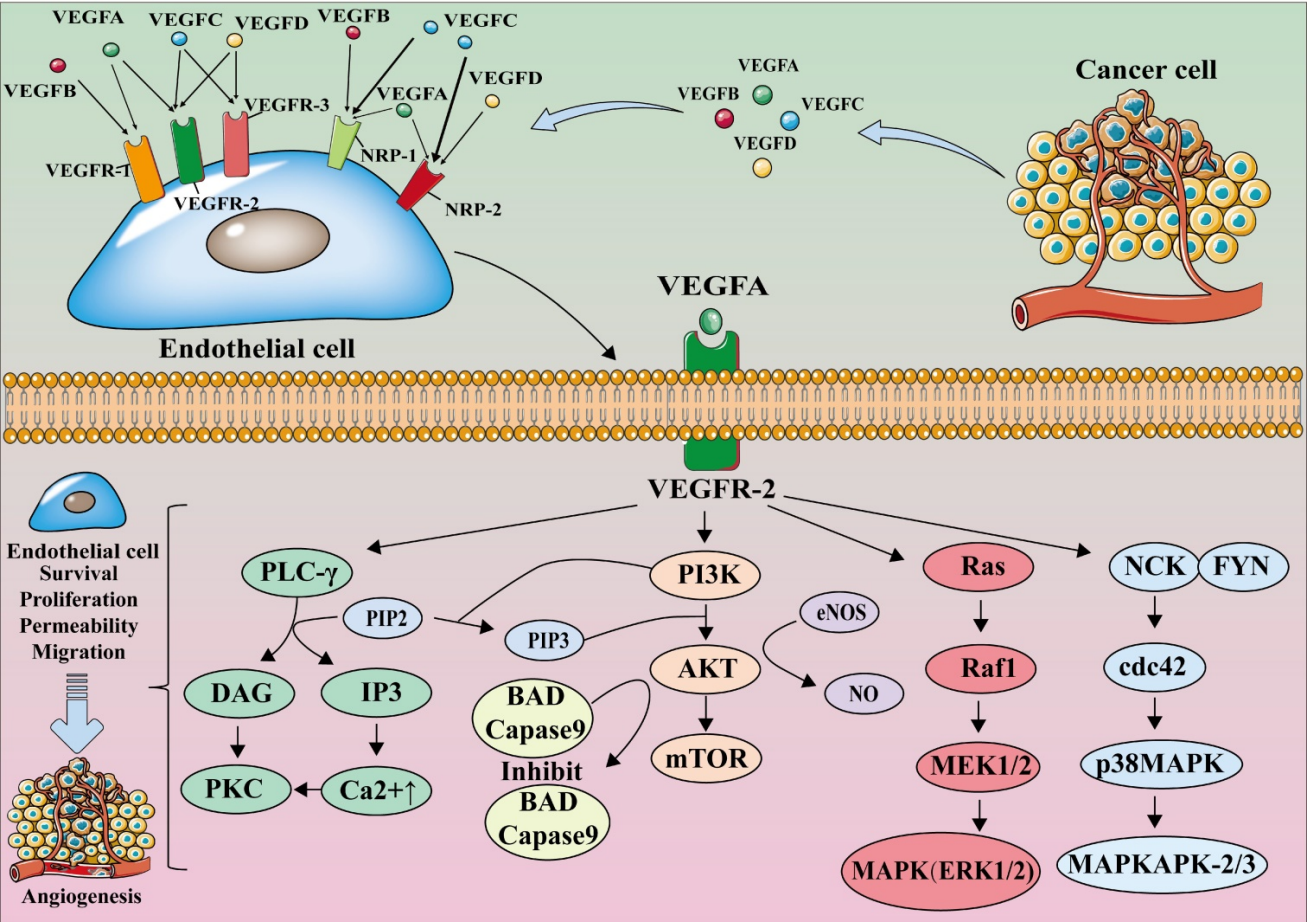
** The VEGF-VEGFR signaling pathway.** VEGF-A binds to VEGFR-2 for dimerization, autophosphorylation, and activation leading to endothelial cell survival, proliferation, permeability, and migration. mTOR, mammalian target of rapamycin; MAPK, mitogen-activated protein kinase; MEK, MAP kinase kinase.

VEGF-A binds to VEGFR-2 and activates phospholipase C γ (PLC-γ). Activated PLC-γ can hydrolyze the membrane component phosphatidylinositol-4,5-bisphosphate (PI(4,5)P2) to produce inositol triphosphate (IP3) and diacylglycerol (DAG). IP3 induces intracellular Ca^2+^ release and improves vascular permeability. Ca^2+^ causes protein kinase C (PKC) to bind and polymerize to the plasma membrane, which is activated by DAG; PKC can also act as an upstream activator of the Raf1-MEK1/2-ERK1/2 pathway, which is central to endothelial cell proliferation 26. The binding of VEGF-A to VEGFR-2 also phosphorylates phosphoinositide 3-Kinase (PI3K), and the activated PI3K binds to the substrate PIP2 converting it to phosphatidylinositol 3-phosphate (PIP3). PIP3 induces serine/threonine-specific protein kinase (AKT) phosphorylation, which activates endothelial nitric oxide synthase (eNOS) to produce nitric oxide (NO) and induces endothelial cell proliferation and migration 27, 28 **(Figure 1)**.

VEGF-B mainly binds to VEGFR-1 and NRP-1, and plays an important role in tumor angiogenesis and in the improvement of ischemic conditions 29, 30. VEGF-C and VEGF-D mainly bind to VEGFR-3 and participate in lymphangiogenesis 30, 31. VEGF-D is associated with tumor metastasis to regional lymph nodes 32-34. Furthermore, PIGF mainly binds to VEGFR-1 and regulates the growth and maturation of blood vessels by regulating endothelial cell and parietal cell proliferation 35 **(Figure 1)**.

### 2.2 Non-angiogenic function: role of VEGF in the functions of TME cell components in NSCLC

#### 2.2.1 VEGF and cancer cells

Evidence suggests that VEGF acts in tumors not only by promoting angiogenesis but also by directly working on cancer cells 36. VEGF can promote tumor development and progression by interacting with receptors expressed on tumor cells through autocrine and/or paracrine mechanisms 37. In addition to tyrosine kinases, NRPs can regulate the function and transportation of growth factor receptors and integrins, thus playing a crucial role in mediating VEGF action on tumor cells 37. Autocrine VEGF in lung cancer has been shown to activate mitogen-activated protein kinase (MEK)/extracellular signal-regulated kinase (ERK) and PI3K/AKT signaling pathways to promote cell proliferation in NSCLC; additionally, NRP1 plays a central role in regulating VEGF-driven NSCLC cell proliferation 38. However, studies showed that blocking endogenous VEGF with bevacizumab, the VEGF antibody, did not inhibit NSCLC cell line growth, suggesting that VEGF alone does not maintain lung cancer cell proliferation in vitro 39, 40. We believe that the development of NSCLC is caused by a combination of factors in the TME. Therefore, in a single cell line without tumor angiogenesis or TME, blocking VEGF alone does not effectively inhibit tumor cell growth. it is likely that VEGF-VEGFR-targeted therapy also acts on the TME to reverse the immunosuppression therein, thus inhibiting tumor growth. Therefore, we infer that combining immunotherapy with VEGF-VEGFR-targeted therapy may exert better therapeutic effects on NSCLC.

#### 2.2.2 VEGF and T cells

Tumor cells evade immune defence by suppressing T cell function, for example, by upregulating the expression levels of T cell checkpoints 41, 42. VEGF-A enhances the expression of PD-1 and other suppressive checkpoints, such as CTLA-4, on the surface of T cells, and suppresses the activity of CD8^+^ T cell, manifesting as a progressive state of dysfunction leading to the blocking of the effector function of T cells 43-45. The inhibitory functions of checkpoints could be reversed by anti-angiogenic agents, as when VEGF is blocked, tumor blood vessels decrease and tumor tissue hypoxia is induced, which in turn activates hypoxia-inducible factor (HIF-1α), and promotes cytokine production to activate CD8^+^ T cells 43-45. However, recently, studies have reported that tumor hypoxia, angiogenesis, and immunosuppression can modulate each other, thus promoting tumor progression and compromising the clinical effectiveness of antitumor therapy 46.

**Figure 2:**
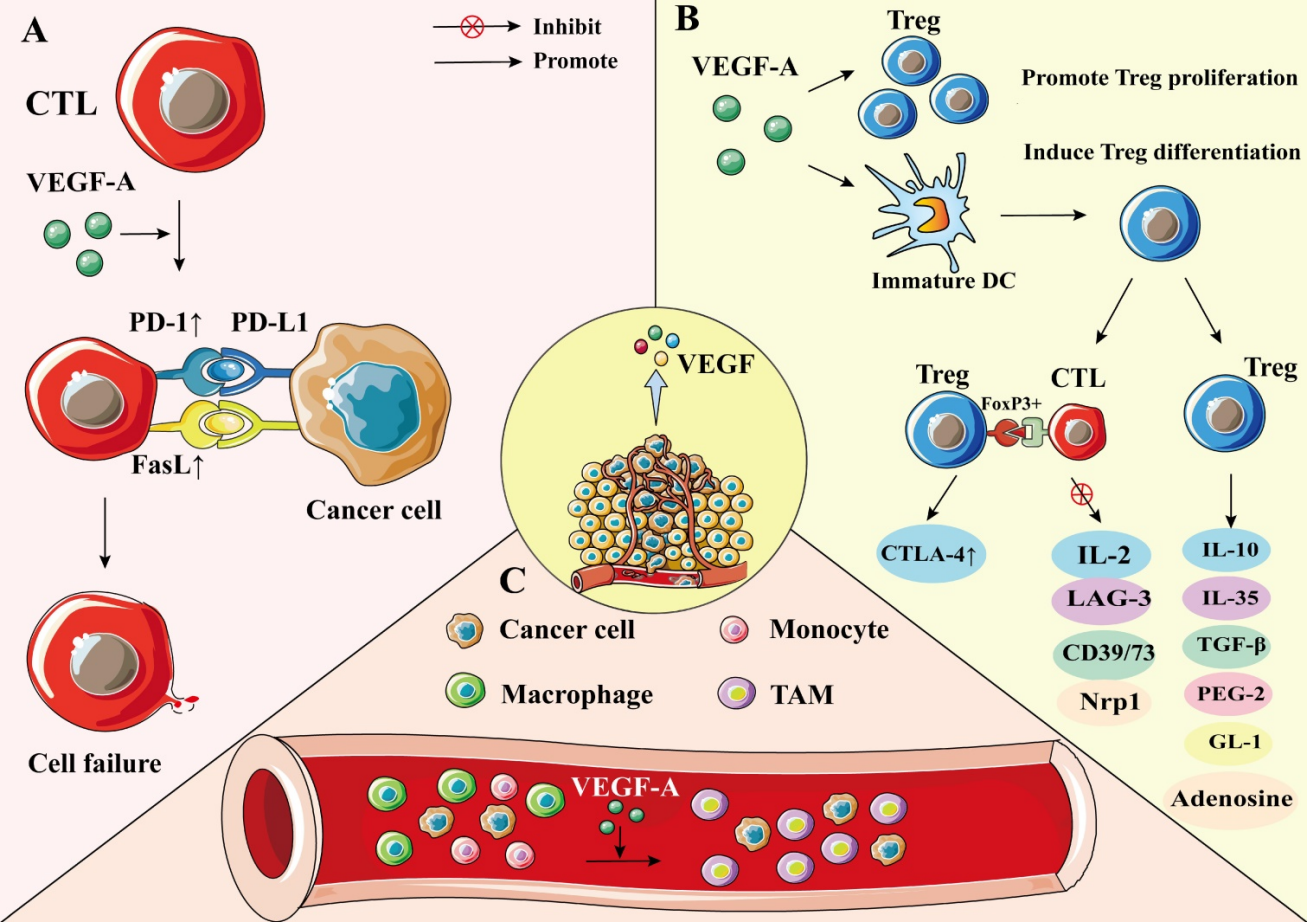
** The effect of VEGF on CTL, Treg, and TAM. A)** VEGF-A enhances the expression of PD-1 and FasL on CTL, thereby promoting CTL cell failure and leading to immunosuppression. **B)** VEGF-A can make Treg recruited in the tumor microenvironment and increase the number of Treg. VEGF-A can induce Treg differentiation by producing immature DC. Treg cells inhibit the activation of target cells through FoxP3^+^ direct contact; up-regulate the expression of CTLA-4; inhibit the expression of IL-2, LAG-3, CD39/73, and NRP-1; or cause immunosuppression by producing soluble immunosuppressive molecules. **C)** Under the action of VEGF-A, monocytes/macrophages in the blood are gathered around tumor cells and differentiate into TAM. The M2 cells in TAM can promote tumorigenesis and development.

Furthermore, VEGF can directly inhibit T cell function. Continuous infusion of recombinant VEGF in non-tumor patients resulted in a decrease in the number of T cells and the ratio of T to B cells in lymph nodes and spleens 47. Additionally, recombinant VEGF-infused mice showed smaller thymus compared with control group mice infused with phosphate-buffered saline, indicating the immune suppressing quality of VEGF 48. In another study, inhibiting VEGF activity by endostatin, a VEGF specific antibody, enhanced infiltration of mature CD8^+^ T cells and reduced the number of immunosuppressive cells in tumors 49. VEGF-A directly inhibits T cell proliferation and cytotoxic activity through VEGFR-2 in advanced ovarian cancer 50. In addition, VEGF-A has been shown to induce the expression of the transcription factor thymocyte selection-associated high mobility group box (TOX) in T cells, which drives a depletion-specific transcriptional program in T cells. In the case of PD-1 blockade resistance, combined blockade of PD-1 and VEGF-A activities restores the anti-tumor function of T cells, resulting in better tumor control 51 (**Figure 2A**).

In addition to directly regulating T cell function, VEGF also can suppress T cell function by regulating Fas ligand (FasL) levels, which is upregulated by VEGF-A in the TME 52, 53. FasL is expressed on the surface of T cells and in tumor endothelium; however, it is not seen in a normal vascular system 48, 54. In human tumors, the expression of FasL in endothelial cells leads to the loss of CD8^+^ T cells 48, 54. In mice, endothelium-secreted FasL suppresses the infiltration of T cells in tumors, thus promoting tumor growth 48, 54 (**Figure 2A**).

#### 2.2.3 VEGF and Treg

Treg cells are a major subset of CD4^+^ T cells. The characteristic phenotype of Treg cells is CD25^+^ CD4^+^ FoxP3^+^ T cell 55, 56. Some preclinical and clinical studies have shown that Treg cells are one of the most common immunosuppressive cell types in tumors 57, 58. They hinder immune surveillance against cancer in healthy individuals, prevent patients with tumors from producing effective anti-tumor immunity, and cause the occurrence and development of various malignant tumors, including NSCLC 59. Treg cells negatively regulate immune response through two mechanisms: (1) by direct contact to inhibit the activation of target cells, such as directly up-regulating the expression of CTLA-4 through FoxP3^+^, thus inhibiting the expression of IL-2, lymphocyte activating protein 3 (LAG-3), CD39/73, and NRP-1 to induce immunosuppression 60, 61, and (2) through a humoral and cytokine secretion mechanism, which involves inducing the production of soluble immunosuppressive molecules, such as IL-10, IL-35, transforming growth factor-β (TGF-β), adenosine, prostaglandin E-2 (PGE-2), or galactose lectin-1 (Gl-1) 60, 61.

In cancer patients, VEGF-A expression was positively correlated with levels of intratumoral Tregs 62. VEGF-A can promote Treg differentiation by inducing immature dendritic cell (DC) numbers 50. Additionally, VEGF-A can directly regulate Treg recruitment in the TME by binding with VEGFR2, which in turn stimulates the proliferation of Treg cells, and enhances the immunosuppressive function 50, 63, 64. Terme et al. reported that tumor-bearing mice show higher expression levels of VEGFR-2 on Treg cells than healthy mice, and that neutralizing VEGFR-2 by specific antibodies can reduce the proportion of Tregs in the spleen of nude mice, indicating that VEGFR-2 signaling plays an important role in regulating Treg functions 65. Furthermore, VEGF-A promotes the infiltration of Treg cells in the TME by binding to NRP-1 66. When the NRP-1 gene was knocked down in T cells, the number of Treg cells decreased, whereas the number of CD8^+^ T cells increased (**Figure 2B**) 66.

#### 2.2.4 VEGF and TAM

TAMs are dynamic cells with multiple polarization states; they are important mediators of cancer development and progression 67. TAMs are present in all stages of tumor development, making them the most abundant immune cells in the TEM 68. There are two types of TAMs, M1 and M2 phenotypes. The M1 phenotype exhibits antitumor effects, whereas M2 is involved in promoting tumor progression 69. TAMs cause immunosuppression by producing cytokines, chemokines, growth factors, and triggering the release of suppressive immune checkpoint proteins in T cells 70. Hwang et al*.* showed that M2 TAM significantly enhanced the expression levels of VEGF-A and VEGF-C of NSCLC cells, whereas M1 TAM only upregulated the expression levels of VEGF-A in NSCLC cells, indicating that TAMs are significantly associated with vascular and lymph angiogenesis, which in turn promotes the progression of NSCLC 5. Additionally, TAM receptors, including Tyro3, Axl, and Mark, bias macrophage polarization toward a pro-tumor M2-like phenotype. These receptors are promising therapeutic targets for tumor-associated macrophages 71.

TAM of the TME is mainly differentiated from bone marrow-derived blood monocytes and monocytic MDSC under the stimulation of tumor cell-secreted cytokines, including VEGF-A, IL-4, and IL-10 68, 72. VEGF-A recruits TAM mainly by binding to VEGFR-1 on the TAM surface 50. TAM expresses PD-L1, which inhibits T cell receptor signaling upon binding to PD-1, leading to T cell inactivation 50. Moreover, M2 macrophages can also release VEGFA, VEGFC, and other growth factors, which may in turn promote cancer progression 70, 73 (**Figure 2C**).

#### 2.2.5 VEGF and dendritic cells

DCs are antigen-presenting cells and have the strongest antigen presentation ability in vivo 74. DCs can produce cytokines and stimulate the differentiation of effector T and NK cells 75, 76. Therefore, DC dysfunction is one of the mechanisms leading to anti-tumor immunodeficiency.

DC can be differentiated from the early stage of hematopoietic progenitor cell (HPC) 77; however, this process may be regulated by VEGF-A 78. Through VEGFR-1, VEGF-A can bind to HPC CD34^+^ cells and inhibit nuclear factor-κB (NF-κB), which is the activator of transcription factors in these cells, thereby inhibiting the differentiation and maturation of DC 78, 79. VEGF can also inhibit DC function by up-regulating PD-1 80. The inhibiton of DC maturation reduces T cell tumor infiltration and exerts immunosuppressive effect 80. Recent findings suggest that VEGF can impair mature DCs' migration ability and immune function through VEGFR-2-mediated RhoA-cofilin1 pathway 81.

Increased immature DCs in cancer patients are associated with increased VEGF levels, which are involved in mediating DC dysfunction 78. Bevacizumab can affect the maturation and function of DCs in vivo by slightly increasing DC numbers and significantly reducing immature myeloid cell numbers 82. Additionally, bevacizumab can reverse the inhibition of VEGF-induced monocyte differentiation into DC in vitro 83.

An investigation of the relationship between VEGF and DC cells using a mouse model found that recombinant VEGF significantly altered DC growth and development at relevant concentrations, with a reduction in the proportion of mature DCs in lymph nodes and spleens of mice 47.

**Figure 3:**
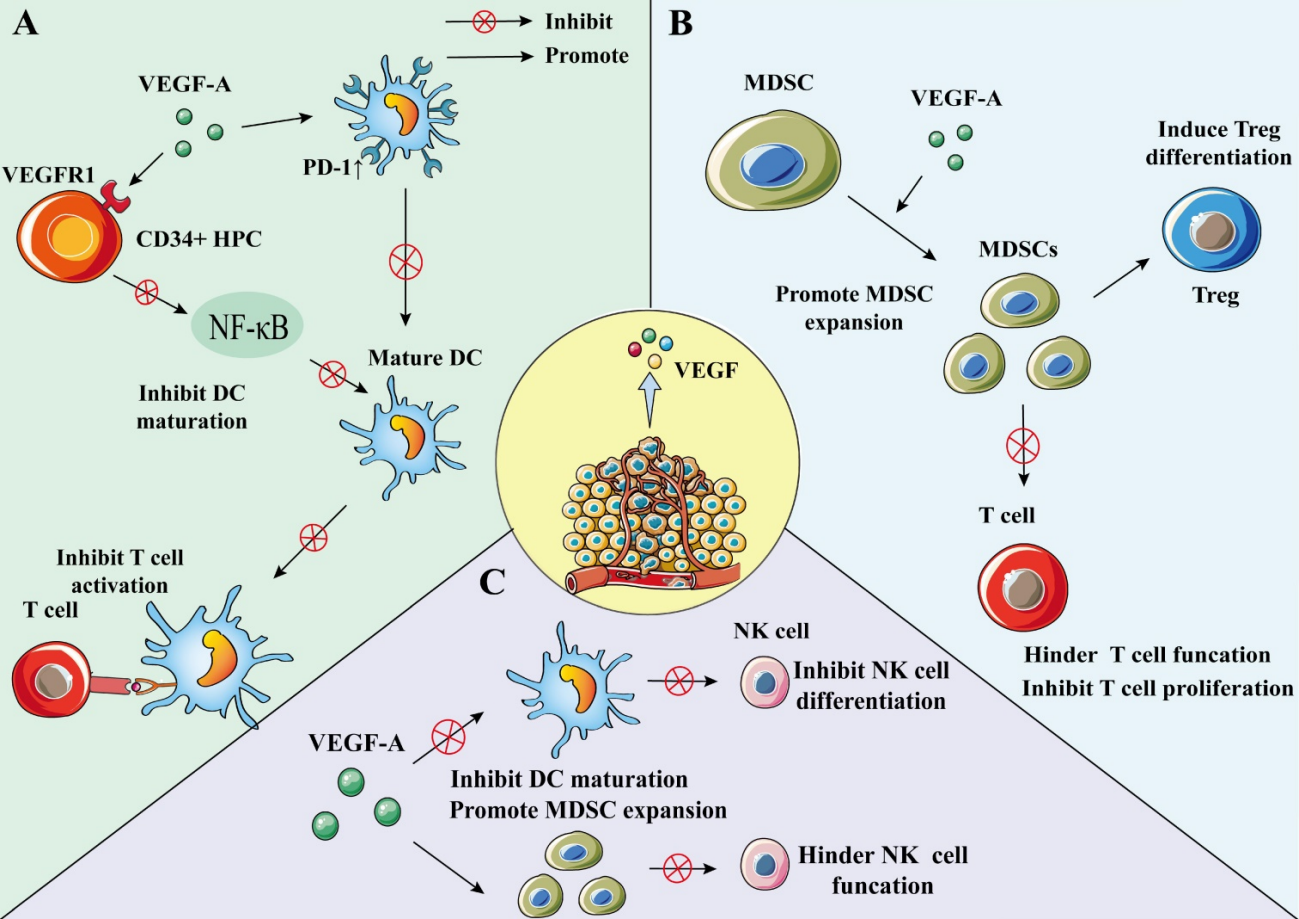
** The effect of VEGF on DC, MDSC, and NK cell. A)** VEGF-A can bind to VEGFR1 on CD34^+^ HPC, inhibit NF-κB, an activator of transcription factors in these cells, and inhibit the differentiation and maturation of DC. VEGF-A can also increase the expression of PD-1 in DC, resulting in a decrease in the number of DCs. To inhibit the activation of T cells by mature DCs. Lead to immunosuppression. **B)** VEGF-A can promote the expansion of MDSC, and MDSC can inhibit the proliferation of tumor-specific T cells and promote the development of Treg. **C)** VEGF-A can inhibit the differentiation of NK cells by inhibiting DC maturation. VEGF can also increase the number of MDSCs and inhibit the function of NK cells.

Furthermore, the results of a clinical study evaluating the relationship between DC infiltration and VEGF expression in NSCLC (132 primary NSCLC treated with surgery) showed that the mean number of infiltrating DCs in the VEGF high expression group was lower than that in the low expression group 84, indicating that VEGF may regulate the infiltration of DC into NSCLC tumor (**Figure 3A**).

#### 2.2.6 VEGF and MDSC

MDSC is a heterogeneous population comprising immature myeloid cells, which are precursor cells of macrophages, DC, or granulocytes features 85. MDSCs are characterized by bone marrow origin, immaturity, and suppression of immune response 85. They can promote tumor cell survival, angiogenesis, tumor cell invading, and metastases 11, 86. Additionally, MDSC induces immune tolerance and can suppress effector T cells and NK cells to induce immune responses 85, 87. Moreover, MDSC could inhibit the growth of tumor-specific T cells and promote the development of Treg cells, which plays a pivotal role in immunosuppression and immune escape 86, 88, 89. MDSCs are also found to be involved in the differentiation of Treg cells. In cancer patients, an increase in MDSC in peripheral blood causes a decrease in mature DCs 90. Many studies have reported that MDSCs play an important role in mediating a variety of tumor-related immunosuppressive functions and tumor immune escape, including NSCLC 89, 91.

VEGF-A is a factor that can promote the amplification of MDSCs; the use of bevacizumab can inhibit VEGF function and MDSC proliferation 50, 92. Research on the effect of lactones on immune cells in the TME showed that MDSC proportion reduced in both the low- and high-dose endostatin groups compared to the control group 49 (**Figure 3B**).

#### 2.2.7 VEGF and natural killer cells

NK cells are a subgroup of cytotoxic innate lymphoid cells in the innate immune system and have a unique killing effect on tumor cells 93. VEGF can inhibit the differentiation of NK cells by inhibiting DC maturation 78, 94. Furthermore, VEGF can increase the number of MDSCs and inhibit NK cell function, in turn leading to the phenomenon of immune escape 89, 95, 96.

Studies have shown that NK cells can secrete VEGF-A under hypoxic conditions, which is a characteristic of the TME 97. Under such hypoxic conditions, VEGF secretion is a transient phenomenon because when NK cells return to peripheral blood, this phenomenon can be reversed 97, 98 (**Figure 3C**).

In summary, VEGF not only promotes tumor growth by promoting angiogenesis but also acts on various immune cells in the TME, which leads to immunosuppression. Therefore, in treating NSCLC, the selection of VEGF-VEGFR-targeted drugs can inhibit tumors from two aspects.

## 3. VEGF-targeted therapy and immunotherapy for NSCLC

### 3.1 VEGF/VEGFR inhibitors and antibodies

VEGF is overexpressed in NSCLC; the expression levels are higher in the tumorous than the surrounding normal lung tissue 99. The high expression of VEGF is related to tumor recurrence, low survival rate, metastasis, and death 99, 100. VEGF is essential for tumor growth and immunosuppression. Therefore, targeted drugs that inhibit the VEGF pathway, such as anti-VEGF monoclonal antibodies and tyrosine kinase inhibitors (TKIs), are used for NSCLC treatment.

Over the past few decades, several VEGF inhibitors have been approved for NSCLC treatment. Among them, bevacizumab, a humanized monoclonal immunoglobulin G 1 (IgG1) antibody that can bind to VEGF-A, has shown good efficacy in treating NSCLC 101. Studies show that chemotherapy combined with bevacizumab prolongs progression-free survival (PFS) and overall survival (OS) in patients with NSCLC compared to chemotherapy alone 23. In addition to combination therapy with chemotherapeutics, bevacizumab has also shown encouraging effects in combination with immunotherapy 80. Data from several clinical trials involving the combination of several immune checkpoint inhibitors and bevacizumab show that when combined with immunotherapy, bevacizumab can prolong survival rate 80. Bevacizumab is also approved by the European Medicines Agency and the United States Food and Drug Administration as first-line treatment for advanced, metastatic, or recurrent NSCLC.

Another VEGF targeting antibody, ramucirumab, is a complete human IgG1 antibody, which can block the interaction between VEGFR-2 and VEGF ligand 102, 103. Ramucirumab functions by inhibiting its signaling pathway by selectively binding to VEGFR-2 104. Additionally, it blocks the activation of VEGFR-2 by ligands other than VEGF-A, including VEGF-C and VEGF-D 105.

Nintedanib is a small molecule TKI 106. In a phase III clinical study, the differences between patients with NSCLC treated with docetaxel with and without nintedanib were evaluated. PFS was significantly longer in patients treated with nintedanib than those in the placebo group 107. Moreover, the OS of docetaxel plus nintedanib was significantly longer than that of docetaxel plus placebo 107.

Furthermore, some other TKIs targeting the VEGF receptor pathway have been tested in clinical trials. For example, lorlatinib has shown clinical activity in patients with advanced NSCLC 108. Another phase II trial showed significantly longer PFS in some patients with NSCLC treated with fruquintinib 109.

### 3.2 Immune checkpoint inhibitor

The immune checkpoint is an immunomodulatory protein that can cause immunosuppression. Antibodies that block the receptors of cytotoxic T lymphocyte-associated antigen-4 (CTLA-4) and programmed cell death protein (PD-1) or its ligand PD-L1 have been approved for clinical use 110. PD-1 and PD-L1 are mainly expressed in immune cells, such as NK cells, DC, CD4^+^, and CD8^+^ T cells 111-113. PD-1 interacts with its ligand PD-L1 to inhibit T cell activation and proliferation, leading to immune escape 114. Significant correlations were observed between the expression levels of PD-L1 and the expression levels of angiogenic factors, such as VEGFA and HIF-1α 115. CTLA-4 is an extracellular surface protein that can control immune suppression, mainly by stimulating T cell receptors 116.

The immune checkpoint inhibitors currently approved as first-line therapy fall into three main categories: Anti-PD-1, Anti-PD-L1, and Anti-CTLA-4. The main inhibitors used for first-line treatment of NSCLC include pembrolizumab 117-119, nivolumab 120, toripalimab 121, sintilimab (Anti-PD-1) 122, atezolizumab 26, 123, durvalumab (Anti-PD-L1) 124, 125, and lpilimumab (Anti-CTLA-4) 120, 126.

VEGF-A inhibits immune activation and induces immunosuppression by affecting various immune cells in the TME. Therefore, immune escape can be suppressed by inhibiting the effect of VEGF, and then combining immune checkpoint inhibitors to treat NSCLC. First, anti-angiogenic drugs can normalize tumor blood vessels and cause tumor immune cells (such as tumor-infiltrating lymphocytes) to increase in NSCLC. Immune checkpoint inhibitor can relieve the inhibition of PD-1 and PD-L1 on T cells, and the synergistic effect of the two shows better therapeutic effects on solid tumors 127. A clinical trial of anti-VEGF drugs combined with immune checkpoint inhibitors is currently underway (NCT04340882).

### 3.3 Vaccines and cell therapy

Cancer vaccines are an active immunotherapeutic intervention against malignancies. Their intended function is to bypass immune tolerance in the TME, thereby suppressing tumor survival. A new antigenic vaccine reported for the treatment of NSCLC combines NEO-PV-01 with PD-1 blockade; it induces T cells with cell-killing effects and has the ability to translocate to tumors 128. A Phase II trial showed the benefit of TG4010 (MVA-MUC1-IL2) vaccine in combination with first-line chemotherapy for advanced NSCLC 129.

Additionally, a clinical trial on the safety and feasibility of CRISPR-edited T cells in patients with refractory NSCLC has been completed 130. Another type of CAR-T cell therapy involves reprogramming the patient's T cells and infusing the modified T cells into the patient's body to attack the cancer cells. However, compared to malignant blood diseases, CAR-T cells have shown limited success in treating solid tumors, including NSCLC 131. Floris Dammeijer et al. found that tumor vaccines and cellular immunotherapy improved OS and PFS in patients with NSCLC, and that cellular immunotherapy was more effective than tumor vaccines 132. Combining VEGF-VEGFR-targeted therapy with a cancer vaccine to bypass immune tolerance in the TME may surpass the expectations of its effectiveness. Nevertheless, more preclinical and clinical studies will be needed to demonstrate this in the future.

### 3.4 Combining VEGF/VEGFR inhibitors and immunotherapy for NSCLC treatment

#### 3.4.1 Preclinical studies

A preclinical study in a human lung adenocarcinoma xenograft model showed that bevacizumab improves the antitumor effect of cytokine-induced killer (CIK) cell transfer therapy 133. The CIK cells in the single treatment group showed no significant antitumor activity compared to the control group, while bevacizumab inhibited tumor growth and reached statistical significance 133. CIK cell therapy combined with VEGF inhibitor therapy showed significant antitumor activity compared to other groups 133. Similar studies in mouse models have also demonstrated that recombinant human endonuclease improves the therapeutic effect of employing CIK cells in lung cancer, revealing the mechanism of the antitumor effect of VEGF inhibitors in combination with immune cell therapy 134.

A study evaluating the synergistic effect of small-molecule tyrosine kinase inhibitor (bosutinib) and tumor vaccine showed that cabozantinib treatment combined with therapeutic tumor vaccine improved the proliferation and function of T cells 135. Tregs from mice treated with cabozantinib alone had a significantly lower ability to regulate CD4^+^ T cell proliferation than Tregs from control mice 135. Moreover, the regulatory ability of Tregs in mice treated with tumor vaccine alone reduced significantly 135. Surprisingly, the combination of cabozantinib and tumor vaccine application eliminated Treg function, as the CD4^+^ T cells of the mice with Treg were not significantly different from those of mice without Treg 135. Additionally, mice treated with cabozantinib combined with tumor vaccines showed a significant increase in both CD3^+^ lymphocyte and CD8^+^ T cell infiltration, indicating an increase in tumor killing effects 135. Other investigators have reported that a low-dose of anti-VEGFR2 antibodies can reprogram the immunosuppressive TME in a manner that enhances anti-cancer vaccine therapy 136.

In 2017, a Japanese research team reported a study using a mouse model, contributing new data on anti-tumor immunotherapy combining VEGF inhibitor (sunitinib) with agonist antibody of death receptor-5. Compared with a single therapy, combined therapy reduced tumor growth rate and increased the number of CD4^+^ Foxp3^-^ and CD8^+^ T cells in patients with tumor 137.

Furthermore, VEGFR combined with T-lymphocyte adoptive transfer also showed potential clinical significance in cancer treatment. A preclinical study showed that adoptive transfer of human VEGFR-1 specificity with chimeric antigen receptor (CAR)-modified T lymphocytes (V-1CAR) can delay tumor growth and formation in mice 138. The results showed that in a mouse model of NSCLC cell A549 xenograft in NOD-SCID BALB/c mice, the growth of A549 xenografts was inhibited by V-1CAR modified T lymphocytes 138.

Moreover, a recent study found that anti-PD-L1 antibody combined with anti-VEGF antibody treatment inhibited tumor growth and increased CD8^+^ T cell infiltration compared with anti-VEGF monotherapy 139. These preclinical results support the idea that treatment efficacy can be improved by combining VEGF inhibitor with immunotherapy.

#### 3.4.2 Clinical studies

Some clinical trials of anti-angiogenic drugs combined with immunotherapy for the treatment of NSCLC are ongoing (**Table 1**).

In a phase III study (NCT02366143), to examine whether VEGF blockade enhances the efficacy of immunotherapy, atezolizumab was added to the combination of bevacizumab and chemotherapy treatment. At the time of data truncation, in the intent-to-treat wild-type (ITT-WT) population, the median PFS of the group with bevacizumab and atezolizumab was significantly longer than that of the group without atezolizumab 140. Furthermore, after 6 months of treatment, the PFS rate of the group with bevacizumab and atezolizumab was higher than that of the group without atezolizumab 140. In the Teff-high WT population, the median PFS was significantly longer in the group with atezolizumab than in the group without atezolizumab 140. Moreover, similar results were observed for OS in the ITT-WT population. However, the rate of objective response was not confirmed, and there were more data for the group with atezolizumab than for that without atezolizumab; the results were similar in the Teff-high WT population 140.

In a phase I study (NCT01454102), the efficacy of nivolumab in combination with bevacizumab was compared to the efficacy of nivolumab alone in patients with advanced NSCLC. The results showed that a higher proportion of subjects were progression-free and survived at 24 weeks in the nivolumab plus bevacizumab group than in the nivolumab monotherapy group. Moreover, the objective response rate (ORR) of nivolumab plus bevacizumab and nivolumab alone arms was 16.7% and 23.1%, respectively.

In another phase I study (NCT02443324), the effectiveness and safety of ramucirumab and pembrolizumab were evaluated in the treatment of different tumor entities, including NSCLC. The ORR of the treated population was 42.3%, and the median PFS was 9.3 months. However, the treated population did not reach the median OS 141.

A study on pembrolizumab plus paclitaxel plus carboplatin plus with/without bevacizumab in stage IIIB/IV NSCLC is also under evaluation (NCT02039674). The results of pembrolizumab plus bevacizumab showed an ORR of 56%, a PFS of 7.1 months, and a median OS of 16.7 months 142.

Moreover, an open phase IB study evaluating the safety, pharmacology, and initial efficacy of the combination of atezolizumab and bevacizumab in the treatment of advanced solid tumors including NSCLC has been completed. However, the results of the study have not yet been published (NCT01633970).

Many studies are currently ongoing. A phase IB trial has been launched with the aim to evaluate the efficacy of pembrolizumab and nintedanib in the treatment of solid tumors, including advanced NSCLC (NCT02856425), and participants are being recruited. A phase I/II study of the efficacy of nivolumab and ipilimumab combined with nintedanib in NSCLC has been initiated, but no results have been published so far (NCT03377023). Additionally, a Phase I/II trial evaluating the feasibility and safety of nintedanib in combination with nivolumab in pretreated patients with advanced or metastatic adenocarcinoma histologic NSCLC has been initiated and is currently recruiting participants (NCT04046614).

Overall, the combination of immunotherapy and VEGF-VEGFR-targeted therapy for NSCLC has yielded encouraging results for such treatment. However, for best therapeutic results, the effects of different anti-angiogenic medications, drug doses, and timing of the combination of the two drugs need to be addressed in future clinical trials.

## 4. Conclusion and Perspectives

This review evaluated the latest knowledge on two therapies, VEGF-VEGFR-targeted therapy and immunotherapy, for the treatment of NSCLC. In addition to promoting tumor angiogenesis, VEGF also promotes immunosuppression in NSCLC. The application of a combination of VEGF/VEGFR-targeted therapy and immunotherapy in preclinical and clinical studies has shown good efficacy in the treatment of NSCLC by regulating the immunosuppressive TME. Results from the currently enrolling clinical studies also support that the combination therapy is a promising approach for the treatment of NSCLC. Future preclinical and clinical studies need to address some key questions, such as what are the specific biomarkers in response to the combination therapy? This therapeutic approach targeting the TME certainly has complex biological effects. Combination therapy may further increase this complexity, posing an increased risk of toxicity and the possibility of immune-related adverse events. For example, immunotherapy can sometimes lead to serious immune-related adverse events, and these toxic reactions can usually be resolved by stopping treatment or reducing the dose. Therefore, it is important to optimize the dose, timing of administration, and sequence of administration of VEGF-VEGFR-targeted drug therapy and immunotherapy in future clinical studies. Since the use of VEGF-VEGFR-targeted drugs allows the drugs or immune cells in immunotherapy to reach the tumor site more easily through normalized blood vessels, the dose of immunotherapy drugs can be reduced appropriately in combination therapy.

While the employment of combination therapy offers a promising future for NSCLC treatment, further studies must investigate how these therapeutic strategies interact with each other to regulate the immunosuppressive TME and kill cancer cells. New technologies, such as single-cell sequencing and spatial transcriptomics, may improve our knowledge. These technologies can help us understand the interactions among angiogenesis, cancer cells, and immune cells of the immunosuppressive TME of NSCLC. Meanwhile, we can design new approaches to target angiogenic and immunosuppressive environment for NSCLC treatment.

## Figures and Tables

**Table 1:** Data from clinical trials of anti-angiogenic drugs combined with immunotherapy for the treatment of NSCLC

NCT ID	Relevant Compound(s)	Phase	Outcome Measures	Status	Study Title
NCT02366143	Atezolizumab + Bevacizumab + Paclitaxel + Carboplatin	Ⅲ	PFS, 8.3 (95% CI, 7.7-9.8) OS, 19.2 (95% CI, 17.0-23.8)	Completed	A Study of Atezolizumab in Combination with Carboplatin Plus (+) Paclitaxel With or Without Bevacizumab Compared with Carboplatin+Paclitaxel+Bevacizumab in Participants with Stage IV Non-Squamous Non-Small Cell Lung Cancer (NSCLC)
NCT02039674	Pembrolizumab + Paclitaxel + Carboplatin + Bevacizumab	I/II	Objective Response Rate, DLT	Completed	A Study of Pembrolizumab (MK-3475) in Combination with Chemotherapy or Immunotherapy in Participants with Non-small Cell Lung Cancer (MK-3475-021/KEYNOTE-021)
NCT01454102	Bevacizumab + Nivolumab	I	ORR, 16.7% (95% CI, 2.1-48.4%) PFSR, 58.3% (95% CI, 27.0-80.1%)	Completed	Study of Nivolumab (BMS-936558) in Combination with Gemcitabine/Cisplatin, Pemetrexed/Cisplatin, Carboplatin/Paclitaxel, Bevacizumab Maintenance, Erlotinib, Ipilimumab or as Monotherapy in Subjects with Stage IIIB/IV Non-small Cell Lung Cancer (NSCLC) (CheckMate 012)
NCT01633970	Atezolizmab + Bevacizumab	I	DLT	Completed	A Study of Atezolizumab Administered in Combination with Bevacizumab and/or with Chemotherapy in Participants with Locally Advanced or Metastatic Solid Tumors
NCT02443324	Ramucirumab + Pembrolizumab	I	DLT, DCR, Objective Response Rate	Active, not recruiting	A Study of Ramucirumab Plus Pembrolizumab in Participants with Gastric or GEJ Adenocarcinoma, NSCLC, Transitional Cell Carcinoma of the Urothelium, or Biliary Tract Cancer
NCT02856425	Pembrolizumab + Nintedanib	I	MTD	Recruiting	Trial of Pembrolizumab and Nintedanib
NCT04046614	Nintedanib + Nivolumab	I/II	PFS, ORR	Recruiting	Feasibility and Safety of Nintedanib in Combination with Nivolumab in Pretreated Patients with Advanced or Metastatic NSCLC of Adenocarcinoma Histology
NCT03377023	Nivolumab + Ipilimumab + Nintedanib	I/II	MTD, DCR, Objective Response Rate	Active, not recruiting	Phase I/II Study of Nivolumab and Ipilimumab Combined with Nintedanib in Non-Small Cell Lung Cancer
NCT03689855	Ramucirumab + Atezolizumab	II	ORR OS PFS	Active, not recruiting	Ramucirumab and Atezolizumab After Progression on Any Immune Checkpoint Blocker in NSCLC
NCT00828009	Bevacizumab + Tecemotide	II	PFS, 14.9 (95% CI, 11.0-20.9) OS, 42.7 (95% CI, 21.7-63.3)	Completed; results published	BLP25 Liposome Vaccine and Bevacizumab After Chemotherapy and Radiation Therapy in Treating Patients with Newly Diagnosed Stage IIIA or Stage IIIB Non-Small Cell Lung Cancer That Cannot Be Removed by Surgery
NCT02574078	Bevacizumab + Nivolumab	I/II	PFS, 6.7 (4.1-NA) OS, 30.8 (8.8-NA)	Completed; results published	A Study of Nivolumab in Advanced Non-Small Cell Lung Cancer (NSCLC)
NCT02681549	Pembrolizumab + Bevacizumab	II	BMRR, ORR	Recruiting	Pembrolizumab Plus Bevacizumab for Treatment of Brain Metastases in Metastatic Melanoma or Non-small Cell Lung Cancer
NCT03527108	Nivolumab + Ramucirumab	II	DCR, ORR, PFS	Recruiting	Nivolumab Plus Ramucirumab in Patients With Recurrent, Advanced, Metastatic NSCLC
NCT02572687	Ramucirumab + MEDI4736	I	DLT, ORR, DCR	Completed	A Study of Ramucirumab (LY3009806) Plus MEDI4736 in Participants With Advanced Gastrointestinal or Thoracic Malignancies
NCT03786692	Carboplatin + Pemetrexed + Bevacizumab + Atezolizumab	II	PFS, ORR	Recruiting	Phase II Randomized Trial of Carboplatin+Pemetrexed+Bevacizumab+/- Atezolizumab in Stage IV NSCLC
NCT02174172	Atezolizumab + PEG-interferon alfa-2a + Bevacizumab	I	RP2D, RECIST	Completed	A Study to Assess the Safety and Tolerability of Atezolizumab in Combination With Other immune-modulating Therapies in Participants With Locally Advanced or Metastatic Solid Tumors
NCT03616691	Atezolizumab+ Bevacizumab	II	ORR, PFS	Unknown	Atezolizumab Monotherapy and Consequent Therapy With Atezolizumab Plus Bevacizumab for NSCLC
NCT03647956	Atezolizumab + Bevacizumab	II	Objective response rate, PFS, TTP, DoR	Unknown	Atezolizumab in Combination With Bevacizumab, Carboplatin and Pemetrexed for EGFR-mutant Metastatic NSCLC Patients After Failure of EGFR Tyrosine Kinase Inhibitors
NCT03713944	Carboplatin + Pemetrexed + Atezolizumab + Bevacizumab	II	PFS, ORR, DCR	Active, not recruiting	Carboplatin Plus Pemetrexed Plus Atezolizumab Plus Bevacizumab in Chemotherapy and Immunotherapy-naïve Patients With Stage IV Non-squamous Non-small Cell Lung Cancer
NCT03836066	Atezolizumab + Bevacizumab	II	Efficacy of atezolizumab in combination with bevacizumab	Active, not recruiting	Atezolizumab Plus Bevacizumab in First-Line NSCLC Patients

PFS: progression-free survival; OS: overall survival; CI: confidence interval; ORR: overall response rate; PFSR: progression-free survival rate; DLT: Dose-limiting toxicity; DCR: disease control rate; MTD: maximum tolerated dose; BMRR: brain metastasis response rate; RP2D: recommended phase II dose; RECIST: response evaluation criteria in solid Tumors; TTP: time to progression; DoR: duration of response*.*
